# Best Practices for Probiotic Research in Athletic and Physically Active Populations: Guidance for Future Randomized Controlled Trials

**DOI:** 10.3389/fnut.2022.809983

**Published:** 2022-03-08

**Authors:** Alex E. Mohr, Jamie Pugh, Orla O'Sullivan, Katherine Black, Jeremy R. Townsend, David B. Pyne, Floris C. Wardenaar, Nicholas P. West, Corrie M. Whisner, Lynne V. McFarland

**Affiliations:** ^1^College of Health Solutions, Arizona State University, Phoenix, AZ, United States; ^2^Research Institute for Sport and Exercise Sciences, Liverpool John Moores University, Liverpool, United Kingdom; ^3^Teagasc Food Research Centre, Moorepark, Ireland; ^4^APC Microbiome Ireland, University College Cork, Cork, Ireland; ^5^Department of Human Nutrition, University of Otago, Dunedin, New Zealand; ^6^Exercise and Nutrition Science Graduate Program, Lipscomb University, Nashville, TN, United States; ^7^Research Institute for Sport and Exercise, University of Canberra, Canberra, ACT, Australia; ^8^School of Medical Science and Menzies Health Institute of QLD, Griffith Health, Griffith University, Southport, QLD, Australia; ^9^Department of Medicinal Chemistry, University of Washington, Seattle, WA, United States

**Keywords:** athletes, physical activity, performance, probiotics, clinical trials, guidelines, study design

## Abstract

Probiotic supplementation, traditionally used for the prevention or treatment of a variety of disease indications, is now recognized in a variety of population groups including athletes and those physically active for improving general health and performance. However, experimental and clinical trials with probiotics commonly suffer from design flaws and different outcome measures, making comparison and synthesis of conclusions difficult. Here we review current randomized controlled trials (RCTs) using probiotics for performance improvement, prevention of common illnesses, or general health, in a specific target population (athletes and those physically active). Future RCTs should address the key elements of (1) properly defining and characterizing a probiotic intervention, (2) study design factors, (3) study population characteristics, and (4) outcome measures, that will allow valid conclusions to be drawn. Careful evaluation and implementation of these elements should yield improved trials, which will better facilitate the generation of evidence-based probiotic supplementation recommendations for athletes and physically active individuals.

## Introduction

Probiotics, defined as living microorganisms that when administered in adequate amounts confer a health benefit on the host, have been widely researched for decades for their efficacy and effectiveness in the treatment or prevention of various diseases (1). One review of the literature from 1977 to 2014 detailed 477 randomized controlled trials (RCTs), encompassing over 15 different types of diseases (typically prevention or treatment of acute intestinal diseases) with >25 different types of probiotic strains or mixtures (2). Since 2014, research has expanded substantially with an increase in both the number of unique probiotic strains studied, and the variety of clinical and wellness applications, including improvement of general health. By 2020, >1,300 RCTs investigating probiotics have been registered, mostly for treating intestinal diseases (78% of the indications) (3). Recently, there is a growing body of probiotic research in relation to recreational and competitive athletes, including investigations in improving gastrointestinal (GI) and respiratory health, exercise performance and recovery, physical fatigue, immunity, and body composition (4).

RCTs are typically the cornerstone of probiotic research and form the foundation for evidence-based substantiation and clinical/practical guidance. While other forms of research may offer support for the use of probiotics in relation to health and safety outcomes, well-performed RCTs (including early phase 2a and 2b designs) provide the strongest causal evidence (5). Investigators have outlined protocols for conducting clinical trials in probiotics (5, 6), however recent challenges in research are necessitating new approaches for probiotic trials. One major development is that the efficacy of probiotics are both strain-specific and disease-specific, so guidelines and practical recommendations on trials using the same strain or mixtures of strains, should be limited to those indications (7).

Human clinical trials with athletes, in comparison to other populations, present additional and unique challenges regarding interpretation, comparison, and, ultimately, the validity of these studies. Contrary to the majority of clinical probiotic research focusing on diseased or unhealthy populations, this type of research often draws from sporting cohorts with typically good health status. Our previous work reviewing probiotic use in athletes reported diverse outcome measurements and different probiotic strains used in RCTs, which limited conclusions on clinical efficacy and real-world effectiveness (4, 8). Here we present recommendations for “best practices” in probiotic clinical research for athletes and other highly active settings including military personnel (i.e., tactical athletes), performing arts, and occupational professions (e.g., in agriculture, mining, and construction industries). In detail, we focus on key elements for an RCT, presenting a framework for future research efforts, including definition and characterization of probiotic interventions, study design factors, study population characteristics, and outcome measures. Our aim is to provide guidance for researchers, clinicians, and other health practitioners who may be interested in probiotics and probiotic-related preparations (i.e., paraprobiotics, synbiotics, and postbiotics) for physical performance-related applications. In addition, we aim to aid health professionals in interpreting the current literature for making informed decisions about probiotic supplementation protocols.

## Probiotic Intervention Clearly Defined

**Figure 1:**
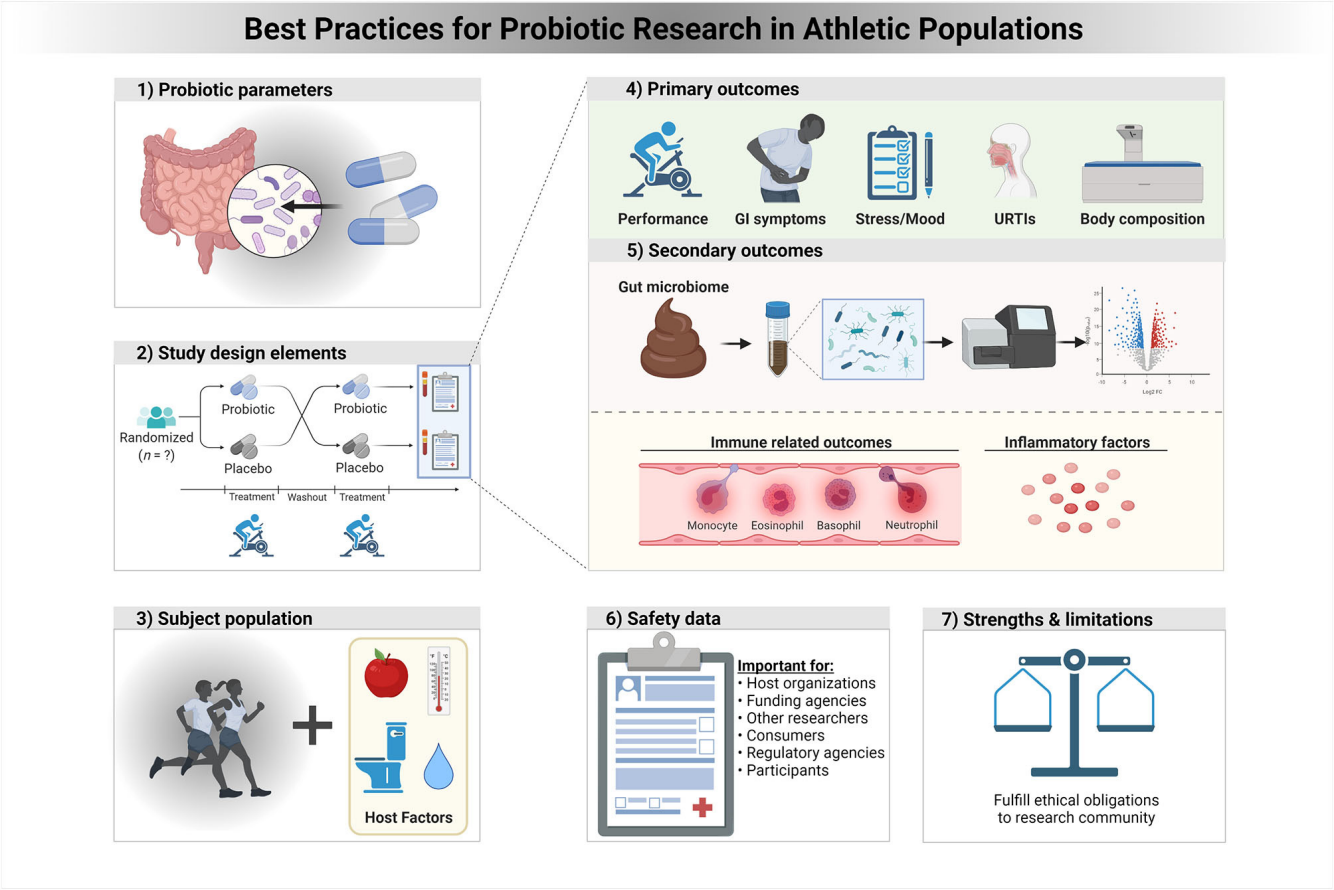
A framework of best practices for probiotic research in athletic populations. In a context specific manner, properly addressing each of these study elements and strategies is expected to improve clinical trials for probiotics and health outcomes in athletes and physically active individuals.

There are several study elements that need to be considered carefully in relation to designing and reporting a probiotic RCT (Figure 1; Table 1). The first element is the choice of the type of probiotic to be tested. Efficacy of probiotics is both strain- and disease/health outcome-specific, and the choice of which strain or multi-strain mixture to use in an RCT must be based on pre-clinical studies that have characterized various factors including: (1) How much of the selected oral probiotic dose survives and reaches the target organ (pharmacokinetic studies) in relation to the outcome of interest (9), (2) whether the mechanisms of action of the probiotic support the health activity of the study outcome, (3) dose and timing, (4) duration and timing of the probiotic intervention, (5) stability and storage requirements of the probiotic (i.e., “shelf-life”), and (6) the additional considerations associated with next-generation probiotics.

**Table 1:** A framework of recommended research study elements and strategies to improve clinical trials for probiotics and health outcomes in athletes and those physically active.

**Study element**	**Required elements**	**Suggested elements**	**Elements done before trial**
1. Probiotic intervention clearly defined	• Strain(s) (genus, species, and strain designations) • Dose • Frequency and duration given • Formulation/mode of delivery	• Dose throughout the study assayed • Timing of consumption	• Selection of appropriate probiotic • Manufacturing processes (lyophilized/ heat-dried/fresh) • Optimal product development for indication (e.g., enteric coated) • Stability/shelf-life defined • Pharmacokinetic studies • Mechanisms of action
2. Study design	• Prospective • Randomization • Use of controls (placebo and/or control group of participants) • Blinding	• Parallel group designs • Crossover designs with adequate a washout period • Use of an adaptive design that can respond to changes in assumptions for power • A run-in phase to determine compliance to the supplement regimen and completion of outcome measures • Follow-up post-intervention • Placebo controls (athletes and/or non-athletes?) • Triple blinded	• Randomization can be employed using methods like block randomization and stratification • Ensure placebos are near identical appearance
3. Subject population	• Age • Sex • Athlete (type of sport or total workload) • Lifestyle factors (fulltime athlete, collegiate athlete, etc.) • Sample size calculations and statistical reporting	• Geography and environmental characteristics (e.g., arid, high elevation) • Dietary factors (dietary assessment or standardized diet) • Medication and health history • Dietary supplement history • Race/ethnicity	• Establish method of subject recruitment and retention. Preferably tested and shown to be effective previously • Establish baseline dietary patterns • Record use of dietary supplements • Cease use of other probiotics, prebioitcs, paraprobiotics, synbiotics, postbiotics, and certain fermented foods (e.g., ~2-3 mo. prior) • Exclude those using confounding medications (e.g., antibiotics, ≤ 3 mo. prior) • No preexisting health conditions that impact gut health (e.g., irritable bowel syndrome)
4. Primary outcomes	• GI symptoms (questionnaires) • Stress reduction and mood profiles • Upper respiratory tract infections • Performance measures	• Immune markers • Body Composition	• Determine how each outcome will be assayed (quantitative data best) from standardized validated assay
5. Secondary outcomes	• Changes in microbiome (pre- vs. post-study, longitudinal samples preferred) • Fecal 16S rRNA, shotgun metagenomics, qPCR	• Immune and inflammatory markers • Plasma and/or fecal metabolomics	• Determine feasibility of microbiome analysis • Establish a standardized protocol for collecting, transporting, processing, and storing microbiome samples
7. Safety data	• Adverse event data from daily diaries	• Distinguish between adverse and serious adverse events	• If known, communicate specific potential side effects to participants
8. Strengths & limitations	• Study-dependent (e.g., laboratory-controlled conditions vs. natural setting) • Mentioned clearly in published work.	• Generalization compared with other studies • Transparent reporting of funding source and potential conflicts of interest	• Register clinical trial with registry (e.g., clinicaltrials.gov) • Implement a RCT checklist • Adhere to established RCT reporting guidelines

*GI, gastrointestinal; rRNA, Ribosomal ribonucleic acid; qPCR, quantitative polymerase chain reaction; RCT, randomized controlled trial*.

### Probiotic Selection

A common misconception is that all probiotics are alike and equally effective. An analysis of 249 RCTs, including 22 different types of probiotics, indicated some strains were efficacious for different diseases, though not all equally effective (10). Thus, the choice of probiotic type is important, should be aligned with the primary outcome of interest, and driven by existing *in vitro*, preclinical and/or human data. For example, should researchers wish to investigate the effects of a probiotic on athlete illnesses and immune function, the choice of which probiotics to use should stem from those that have either; demonstrated the potential to improve immune function via an identified mechanism in animal or *in vitro* model(s) or, preferably, shown effectiveness in other human populations. In more traditional athlete-probiotic research, multiple strains have been identified for their immune boosting and GI health properties, however, some early-phase work is showing potential probiotic benefit for augmenting exercise metabolism, improving nutrient absorption and body composition, and increasing neurotransmitter synthesis (4).

### Linking Mechanisms of Action of Probiotics to Effects on Host Systems

Exogenous supplementation of probiotics may affect the composition, functionality, and metabolism of the resident gut flora in ways that enhance health (11). However, the mechanisms by which they may affect the health of the host are much wider. For example, probiotics can affect health by competitive exclusion of pathogens, production of short-chain fatty acids, and upregulation of anti-inflammatory cytokines (12). Moreover, probiotics may interact with the immune system *via* gut associated lymphoid tissue (GALT) and intestinal epithelia, thereby impacting immunity and inflammatory states (13). In many research studies, probiotics are seen as the input, and the health outcome as the output, with many studies not fully describing the intervening mechanisms (4). While it is not expected that clinical researchers fully identify and evaluate mechanisms of action in their studies, there should be a basis and rationale for selecting a probiotic strain and study outcome, as well as some attempt to assess these mechanisms in action.

Although a full examination of the mechanisms of probiotics is beyond the scope of the current review, some of the best examples of mechanistic assessment of probiotic supplementation in athletes come from studies investigating their effect on immune health. For example, when investigating the effect of probiotic supplementation on mucosal immunity in athletes, Cox and colleagues selected a strain that had previously shown efficacy in animal models (14). In addition, as well as assessing incidence, duration, and severity of illness as the primary outcome measures, they also assessed salivary IgA and IgA1 and circulatory measures of numerous cytokines. Mechanistic assessment is desirable for several reasons. Such approaches have (although not always) been used in other areas of athletic research. GI symptoms can manifest both at rest and during exercise for several reasons (see below). If probiotics are to be considered as a strategy to reduce them, it is important to fully understand where they are most likely to be of benefit. For example, the mechanisms behind infectious diarrhea are different to exercise-induced diarrhea, and so different probiotics may be more or less beneficial in each circumstance.

Work from *in vitro* and animal research may be pertinent, as well as shorter duration human studies. For example, Jäger et al. assessed amino acid concentrations in the blood following ingestion of a protein bolus with or without a probiotic supplement [5 × 10^9^ colony forming units (CFU) *Lactobacillus paracasei* LP-DG and 5 × 10^9^ *L. paracasei* LPC-S01] using a randomized, double-blind, crossover design in physically active males (15). In addition, proteolytic ability of the probiotic was tested using *in vitro* assays. This type of investigation could better inform longitudinal experimental work where researchers could, for example, evaluate lean body mass and body composition during a training program in conjunction with a protein supplement [e.g., (16)].

Many probiotics have some shared functions. For example, lactic acid-producing bacteria possess varying levels of bile salt hydrolase (BSH) activity which improves GI survival and persistence (17). Understanding the effects of multi-species/strain probiotics may be more complicated. In these formulations, delineating one probiotic strain from another or capturing synergistic effects requires complex study designs. To our knowledge, such work has not yet been conducted in athletes. Regardless, researchers should match mechanisms of actions with relevant outcomes. As an example, if a probiotic strain has evidence of supporting gut epithelial barrier integrity perhaps the investigators of a clinical trial could explore GI symptoms as a primary outcome, and indirect intestinal permeability (e.g., urine lactulose and mannitol) as a secondary outcome. Other proposed mechanisms of action for athletes include production of health-associated analytes, adhesion to the intestinal mucosa, modulation of the immune system, and improved nutrient absorption (4).

### Dose and Timing of the Probiotic

**Figure 2:**
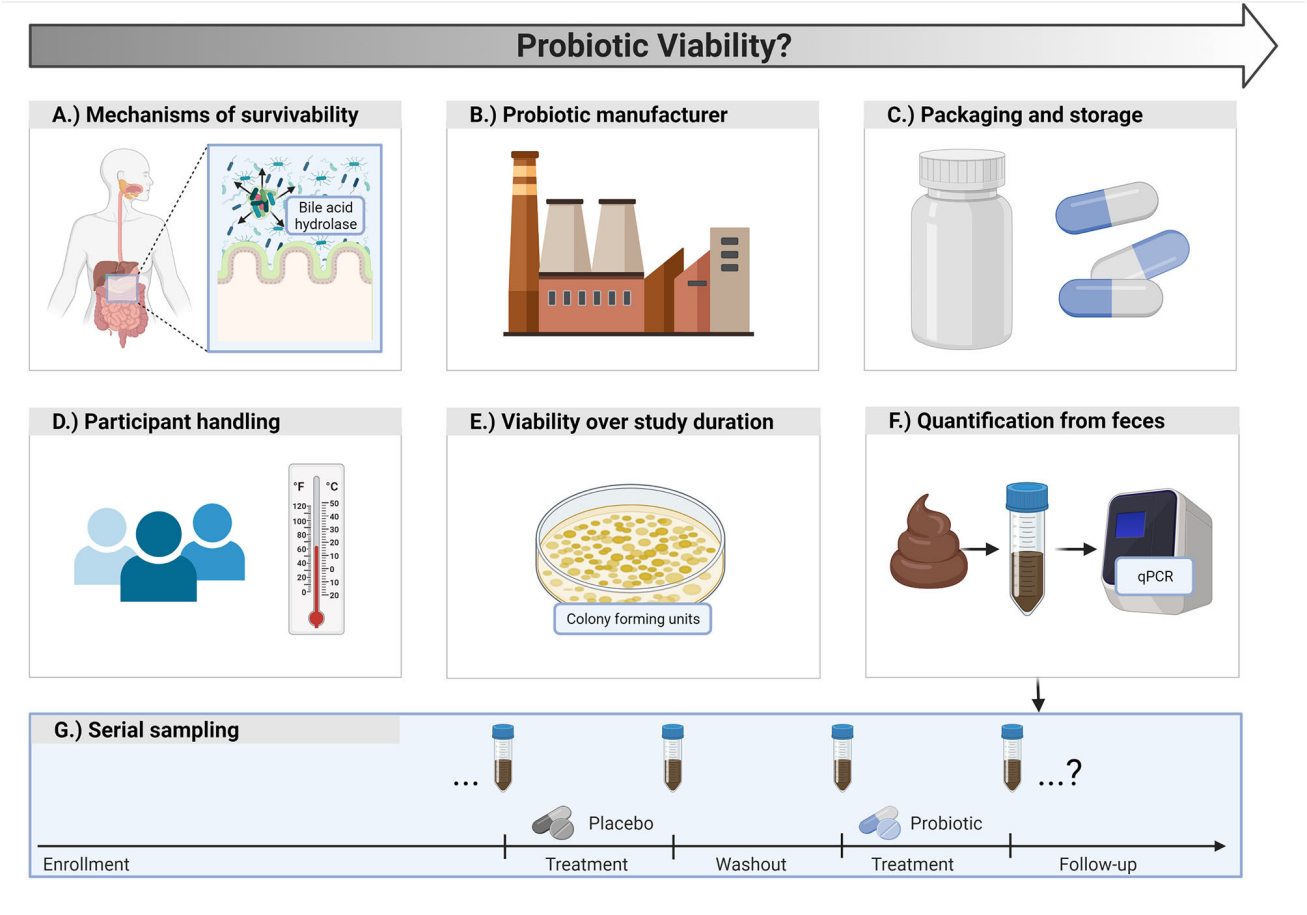
Probiotic viability considerations for clinical research. **(A)** Probiotics should have characteristics that promote survivability in the gut. **(B)** Manufacturers should ensure viable counts of probiotic cells until the end of the shelf life of the product. **(C)** Probiotic packaging and storage conditions are important for viability. **(D)** Participants should be instructed on appropriate handling and storage of the probiotic. **(E)** Viable counts of the probiotic product should be assessed at the start and end of a study to ensure potency is maintained. **(F)** Researchers may consider assessing participant fecal samples by quantifying the probiotic strain of interest *via* quantitative polymerase chain reaction (qPCR) to better gauge survivability. **(G)** Depending on the study design, strain quantification can occur at multiple, strategic time points.

For a probiotic to be effective, it must survive passage from the oral cavity to the target organ (usually the GI tract) and be present at an effective dose within the target. Typically, oral doses of probiotics have ranged from 10^8^ to 10^10^ CFU/g per day. Important factors that influence the probiotic dose at its target include the original oral dose, type of formulation (enteric coated capsules vs. live in food/beverages, or a combination), persistence within the host, colonization at the target organ, manufacturing processes, and stability of the final probiotic product (i.e., shelf-life). Therefore, preserving probiotics in a live and robust state over the entire length of an experimental investigation is paramount (5) (Figure 2). Importantly, the likelihood of surviving GI transit is dependent on the quantity of live cells ingested, as well as self-preserving properties of the microbial strain. Consequently, appropriate production/manufacturing, packaging, handling, and storage procedures adopted prior to ingestion are critical. Finally, timing of consumption may also be important in some instances. For example, recent work has highlighted the improvement of macro/micronutrient absorption, such as protein and iron (15, 16, 18). In two of these studies, the investigators reported the probiotic was consumed with food (15, 16), whereas one did not (18). In these applications, the timing of intake would be helpful to report in future work, and should provide important insights on factors involved in colonization in the gut and nutrient interactions/metabolism. Overall, practitioners should carefully evaluate quality evidence-based guidelines and research materials to inform their clinical advice, and researchers similarly should critically evaluate relevant literature, engage with appropriate professional bodies, and industry/manufacturers willing to collaborate with independent research groups.

### Duration and Compliance of the Probiotic Intervention

The duration of supplementation and adherence to study consumption requirements are critical factors as the typical time for adaptation to the probiotic can be rapid (4). With cessation of probiotic intake, there is a reduction in the amount of probiotic in the colon, and within approximately a week of discontinuing supplementation, the probiotic is no longer detectable in the feces (19, 20). The long-term effects of probiotic administration in athletes over several months or years on gut health, immune function, and rates of illness are unclear, as in most studies the supplementation period was between 4 and 16 weeks (4). Moreover, it is uncertain if there is a habituation effect, where effects could potentially dissipate as the host systems “normalize” to the treatment. Defining post-intervention follow-up periods is also important to document any safety assessment and verify whether the outcome measures persist or disappear with clearance of the probiotic. Ultimately, the recommendation for study duration should depend on the primary outcome measure and the specific strain utilized. The decision on the length of the intervention must be made on a case-by-case basis for both clinicians and researchers addressing individual requirements (clinically), and research opportunities and limitations for academic researchers. Finally, assessment of compliance is an important issue in this population, as large variation in day-to-day compliance with dietary supplementation between athletes has been noted (21).

### Stability of the Probiotic Product

Manufacturers should guarantee the maintenance of a viable count of probiotic cells for trials examining live bacteria until the end of the specified shelf life of the product (22). Probiotic viability throughout the storage period of commercial products can vary substantially (22). Indeed, multiple studies have reported a reduction in live cell counts below the limit stated on the label (23). The decrease in viability of probiotics over the shelf life of a product underscores the importance of selecting probiotic strains with robust stability properties, use of excipients with a low water activity, and ensuring appropriate and validated packaging. In one trial, investigators were forced to exclude the *L. rhamnosus* GG probiotic intervention arm given a significant decline in probiotic counts at the end of the study (declined from 10^9^ to 10^6^ CFU/g), showing the importance of product stability (24). The quantity of viable probiotic cells tested from different sites or products will also depend on methods used for enumeration (22, 25, 26). Assessing the viable count of the probiotic product used at both the initiation and conclusion of a study is recommended to confirm that the potency is maintained (5). For long-term research over several months or more, assessment at one or more mid-points is also prudent. While we acknowledge that non-viable probiotics and probiotic fragments (along with postbiotics) may be sufficient to elicit host responses, we are specifically referring to probiotic (per the formal definition) integrity of the supplement under investigation. Reporting these data as the actual viable count and target viable count of the probiotic can be included in the published work.

### Considerations for Next-Generation Probiotics

Technological advances and deeper research efforts into the human gut microbiome are driving the identification of potential next-generation probiotics. Potential candidates are represented by microbial genera and species that have not been used in the food industry (27). Several have been identified from the human GM through strong associations with improved health status including *Akkermansia municiphila* (28) and *Faecalibacterium prausnitzii* (29). In terms of clinical research, the main questions with these microbes relate to their technological robustness, safety, and effectiveness. Both *A. municiphila* and *F. prausnitzii* are anaerobes and therefore extremely sensitive to oxygen. To avoid the problems with viability and stability, many studies with *F. prausnitzii* have been undertaken with culture supernatants instead of live cells (27). In the first human RCT with *A. municiphila*, measures were employed to better ensure the participants were consuming live cells (30). For example, probiotics or placebos were supplied to the subjects every 2 weeks during follow-up visits, with instructions to take one dose every morning on an empty stomach. Participants were instructed to keep the packages in the freezer compartment of a home refrigerator until time of consumption. A temperature sensor was also provided to all participants to monitor the temperature during transport and home storage (at −20°C). Building such precautions into a protocol, operationalizing them, ensuring participant compliance when necessary, and recording/reporting of protocol or compliance deviations, is highly recommended for these novel probiotics.

Next-generation probiotic studies in humans are beginning to emerge with the primary end points of safety, tolerability, and metabolic parameters. Currently, these remain limited to clinical populations (30). However, there are some potential candidates which appear to have direct application to athletes. The commensal taxa *Veillonella* may metabolize lactate into the short-chain fatty acids acetate and propionate *via* the methylmalonyl-CoA pathway (31). Pre-clinical studies with an isolated strain from marathon runners, *Veillonella atypica*, showed a 13% increase in endurance performance (31). Comprehensive safety and tolerability data (e.g., nausea, flatulence, bloating, cramps, borborygmi, and gastric reflux) will need to be collected during supplementation trials as well as in follow-up periods (e.g., 3 months after intervention cessation) (30). In addition to questionnaires, blood sampling, and clinical examination should be performed to compare clinical parameters with baseline values and a placebo control.

**Key Recommendations:**

- Choose the probiotic strain or strains to be tested on the basis of prior studies of the pharmacokinetics, stability, mechanism of action, and established health outcomes.
- Define the oral daily dose based on survivability to the gut.
- Define the duration and timing of the probiotic intervention based on probiotic persistence, time to observe an effect on the host, and sufficient time for outcome measures to change.
- Maintaining probiotics in a live state with the appropriate dosage over the duration of a study is essential. Researchers should ensure survivability through robust manufacturing processes, packaging and storage, and biochemical properties of the probiotic strains.
- Probiotic viability of the research product can be accessed *via* study participant's fecal samples, using established methodology such as quantitative polymerase chain reactions (qPCR) to quantify the strain under study.
- Clinical investigation of next-generation probiotics may require additional assessment and reporting of technological robustness, safety, and effectiveness.

## Study Design

The ideal study design for testing probiotics is a prospective RCT. Control participants should be provided a placebo of identical appearance, size, and taste. In certain studies, a run-in phase may be a prudent addition to determine compliance to the supplementation regimen and also gauge completion of outcome measures. Where possible, researchers should use triple-blinding where the probiotic treatment is unknown to the participants, research staff that administer it, and the individuals that perform the analyses of the outcomes. This protocol can help prevent bias due to demand characteristics or the placebo effect. Moreover, many probiotic trials may be better suited as parallel group designs to address the key issues of feasibility, concerns of attrition, and the requirement of lengthy wash-out periods. However, probiotic response could be driven by the underlying GM; therefore, some research questions may benefit from crossover trials. Essentially, if GM composition is suspected to drive the probiotic response or mechanism, then crossover may be more relevant for elucidating or confirming those mechanisms. Moreover, cross-over designs have greater statistical power compared to parallel group designs. If a crossover design is used, then the researchers need to ensure the probiotic has left the system, and any potential effects have dissipated from the first treatment to prevent carryover effects. Robust consideration of the research question in relation to intestinal mechanisms, treatments, and outcomes is needed when selecting an RCT design, specifically if parallel vs. crossover designs are merited. Finally, researchers should consider the use of an adaptive design that can respond to changes in assumptions for power (for instance if the underlying upper respiratory tract infection (URTI) rate is lower or higher than expected then the sample size can be changed) (32). These considerations should be weighted by when the study is conducted, as in the case of URTI prevalence during winter vs. summer (32), or possibly in the case of specific athlete populations, focused around the time that URTI incidence is at its highest. Regardless of the study design type, follow-up assessments post-intervention are also encouraged. These elements provide important information on the dissipation of effects and persistence of the probiotic.

**Key Recommendations:**

- Control supplements should be of identical appearance to the treatment and, when feasible, triple-blinding should be used.
- A run-in phase may be indicated to determine compliance to the supplementation regimen and completion of outcome measures.
- Prospective parallel-group RCTs may be better suited for probiotic trials. However, if the researcher is interested in the GM as an influencing factor in relation to the probiotic, a crossover design may be preferred. If a cross-over design is used, the wash-out period must be of sufficient duration (2–3 weeks minimum) to reduce potential carryover effects.
- Use of adaptive designs (where appropriate) that can respond to changes in assumptions for power.
- Where appropriate studies should account for seasonal effects and timing of intensive training and/or competitions.
- Follow-up assessments post-intervention are encouraged with their length dependent on the main outcomes of the study.

## Study Population Characteristics

### Age, Gender, Race/Ethnicity, Geography, and Underlying Symptoms/Disease

Age and gender are important characteristics to consider in athlete probiotic studies as previous literature has largely been limited to males and young adults. A review of probiotic trials in athletes noted the mean age of the participants was below 40 years, with the majority in their 20's (4). Moreover, most of the trials were performed with males or a predominately male sample. However, there have been some studies that have exclusively investigated the effects of probiotics in females (16, 33). Notably, there is some evidence that females and males may not respond to a particular probiotic in the same way. For example, West et al. used a sample of both male and female competitive cyclists and reported that males responded more effectively to *L. fermentum* (PCC) than females (34). The supplementation protocol was the same, including dose and duration. Gender-specific issues need further investigation as many commercial products do not differentiate dose between genders. In addition, some strains may have more attractive features for a certain gender. For example, Axling et al. investigated the probiotic strain *Lactobacillus plantarum* 299v, specifically for its reported benefit of increasing iron absorption in female athletes with low iron stores (ferritin < 30 μg/L) (18). Such gender-specific applications are important considerations for future research efforts.

Race and ethnicity may play an important role in probiotic response including consideration of delivery mechanisms (e.g., milk-based vs. non-milk-based products). Whenever possible, research should be inclusive of all populations and include race and ethnicity covariates in analyses. For example, lactose malabsorption affects ~70% of the world adult population with consumption of milk and milk-based products (35). Many athletes represent minority populations, including those of African, Asian, and Hispanic/Latino decent, who may be particularly vulnerable to food-related GI issues (36). Therefore, selection of probiotic vehicles like yogurt, cheese, and milk should be avoided in athletic populations where lactose malabsorption may be of concern. Even after adjustment for confounding factors such as gender and dietary intake, ethnicity exerts a significant influence on the GI microbiota of individuals (37). While studies are limited, there is evidence to suggest the effectiveness of a probiotic may differ based on ethnicity. A meta-analysis of probiotics in the treatment of acute rotavirus diarrhea in children showed poor therapeutic efficacy in Asian children, but a significant protective effect in Caucasian children (38). Future studies in athletes should therefore provide information regarding participant ethnicity where possible.

Another important consideration for athlete probiotic studies is whether clinical, sub-clinical or sport-induced (e.g., endurance running and GI distress) symptoms or conditions might influence outcomes. As such, it may be useful to use various assessment methods to screen for potential diet-related GI symptoms before enrollment using questionnaires (e.g., Gastrointestinal Symptom Rating Scale), or more quantitative measures such as breath hydrogen to identify malabsorption issues (39). Participants should be screened for underlying symptoms, and/or clinical pathology at recruitment and induction to studies. In a large cohort of elite athletes, 15% reported at least one GI-related symptom rated as “moderately severe” or worse (40). In recreational athletes, up to 10% can present with irritable bowel syndrome that has not previously been diagnosed (41). It is therefore vital that studies record and present data ascertaining to relevant participant characteristics such as GI symptomology, and/or pathology or other clinically relevant information that could confound study outcomes (e.g., asthma/allergies in studies investigating probiotics and immune function).

When athletic performance or respiratory illness are the primary outcomes of a probiotic study, it is important to consider how geography and the environment might interact or confound outcomes. For example, issues such as elevation, exposure to potential allergens, extreme temperatures, and humidity differences all have the potential to impact performance outcomes. Depending on the primary aim of the study, longer durations may be needed to more adequately account for these effects. This requirement is especially prudent for URTI incidence and the possible effects of season. While burdensome, these studies have been successfully implemented in professional athletes and provided valuable data for illness prevention strategies during periods of intensive training and in winter months (42). Moreover, different environments harbor different biomes which may influence the GM of athletes living or traveling to these areas. Investigators should report the location where their study took place and, if appropriate, provide a detailed description of the environmental conditions encountered. This is especially important for athletes who may travel regularly for competitions and training.

### Type of Athlete/Exercise

A wide range of different sports and skill levels have been assessed in probiotic research from recreational to professional athletes (4). There has been a skew toward endurance-based athletes, such as runners (43–47), cyclists (48, 49), and triathletes (50–52), in part due to greater frequency of reported GI complaints. Research studies have addressed other (team) sports including baseball (53), American football (54), rugby (55), and volleyball and soccer (16). The probiotic a researcher may wish to examine in a particular group of athletes should be established based on the mechanism of action and potential benefits of the probiotic. Athletes from different sports may rely more on certain energy systems and have different environmental exposures, dietary intakes, and recovery times which may influence probiotic requirements. In power-based sports like American football or rugby, muscle recovery, and total energy and protein consumption can be markedly different compared to other sporting cohorts (56). For example, using probiotics with increased proteolytic activity to improve protein digestion can promote muscle recovery (15, 57). Probiotics with this activity could be used in trials with more anaerobic-based athletes. On the other end of the spectrum, certain probiotics for endurance athletes can increase absorption and oxidation of carbohydrates to enhance exercise metabolism (48). For secondary outcomes like the GM, athletes exhibit gut microbial profiles that differentially reflect activity level, volume of exercise, and diet (58, 59). Further, host and GM feature differences appear to impact probiotic efficacy, but much is still unknown and, for now, these differences are not predicted by stool-based measures (60).

### Dietary, Supplementation, and Medication Factors

Dietary intakes should be controlled or at least accounted for when conducting supplementation studies including probiotics. Use of condensed metrics such as the Healthy Eating Index (HEI) can be implemented. Such dietary pattern indices are likely to provide greater detail regarding dietary diversity than simply reporting individual nutrients, and can explain some of the variability in GM (61). Validated Food Frequency Questionnaires (FFQ) offer a way to measure global diet over a period of time, and establish dietary patterns which could influence baseline GM (62). For example, FFQ have been validated in the measurement of prebiotic intake (63). Providing information on the participant's habitual dietary patterns could provide a better understanding of the responsiveness of the GM to a prescribed intervention (61). However, completing a baseline dietary pattern assessment does not address any acute changes in diet that might occur during the intervention. The most accurate method to control the diet is to provide a standardized diet in the week before, and throughout, the intervention. However, this approach can be expensive and logistically difficult, especially during longer-term studies. Alternatively, measurement of dietary intakes and reporting these intakes should be included with any intervention.

There are several types of food diaries which can be implemented to detect acute changes in energy or macronutrient intake over the period of an intervention. Generally, a dietary log of 3–4 days is desirable, including a weekend day (64). If this method is used, a weighed food diary should be completed 3–4 days leading up to commencement of a probiotic intervention, and repeated 3–4 days prior to the post-intervention measures. Moreover, participants will need to be provided with instructions on how to complete these food diaries appropriately. As a drawback, food diaries can result in behavior change as participants may feel obligated to improve/alter their diet. Therefore, another option that could be used is multiple 24-h recalls over the course of the study. Preferably, the investigator should use the multiple-pass method, which is an interviewer-administered 24-h dietary recall employing five steps designed to enhance complete and accurate recall and reduce respondent burden (65). Finally, as an option of implementing a combination of methods, investigators could use multiple 24-h recalls and FFQ that focus on assessing the diet without intervening in dietary choices that participants make (i.e., food diaries). Such a method may be time-consuming, but provides a robust representation of recent and current dietary behaviors. Selecting the most appropriate dietary monitoring tool ultimately depends on the nature of the study, the participant population, and feasibility constraints. Data on baseline and post intervention energy, macronutrients including fiber or dietary patterns should be reported in the manuscript.

Dietary supplements are popular among athletes and have the potential to influence the outcomes of a probiotic trial. All dietary supplements should be recorded by the investigators. Other probiotics, prebiotic fibers (e.g., inulin and galactooligosaccharides), and, in some cases, fermented foods (e.g., sauerkraut, kombucha, and kimchi) should be eliminated prior to and during the study. This practice should be followed for 3–4 weeks before the start of the intervention period. Medications present a similar confounding issue, which may be more problematic in some instances including use of antibiotics. This is especially important if an investigator is assessing the GM as an outcome. Participants may need to be excluded in these cases as supplement use may substantially confound the study results and conclusions.

**Key Recommendations:**

- Characteristics of the study participants should be considered at the initial ideation stages of a probiotic intervention. Age, gender, race/ethnicity, and geography can all potentially impact study outcomes.
- The athletic population an investigator is targeting for their study should be established based on the mechanism of action and potential benefits of the probiotic.
- Characteristics of the athletes including sport, competition level, training history, medication usage, and anthropometric data should be reported, and groups matched for potentially confounding factors.
- Dietary intakes (preferably 1 week) prior to and during the intervention need to be reported.
- Dietary intakes should be controlled as much as possible for the duration of the probiotic intervention, and during the placebo or control phase.
- Groups (intervention vs. placebo) should be matched for baseline dietary patterns.
- Dietary supplement use should be recorded or, when appropriate, eliminated (preferably 3–4 weeks) prior to the start of the intervention.
- Athletes taking medications that could interfere with the study outcomes should be screened, and where appropriate, excluded during the recruitment process.

## Primary Outcomes

Study outcomes can typically be categorized as either primary or secondary outcomes, though this is often not discerned in many studies. Primary outcomes are defined as those that have direct application to the target athletic population, use validated measurements, and the focus of effect sizes and power analyses. The primary outcomes of specific interest in athletes and active populations are GI symptoms, stress and mood measures, URTIs, and exercise performance and recovery. Secondary outcomes are more complex and typically describe the potential mechanisms behind the primary outcomes.

### Gastrointestinal Symptoms

**Figure 3:**
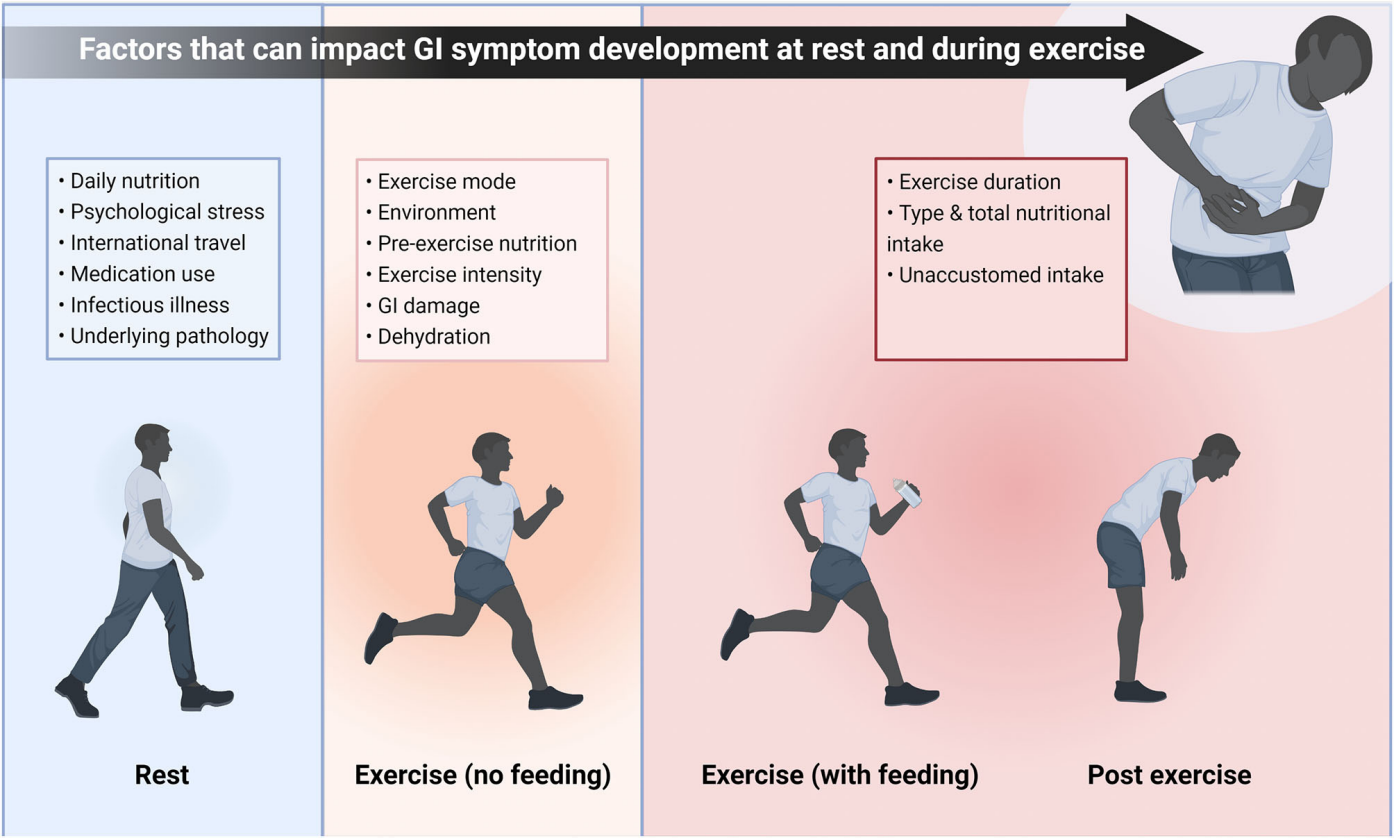
Potential factors that may play a role in GI symptom development either when the athlete is at rest, during exercise with or without peri-exercise feeding, or post exercise. While these factors will not always lead to symptoms, there are a wide range of potential triggers and complaints are likely to be individual. Therefore, there are multiple potential areas probiotics could affect GI symptoms in athletes, broadening the scope for potential research. This schematic also illustrates the large number of confounding factors that should be considered when investigating GI symptoms of athletes. GI, Gastrointestinal.

One of the most extensively researched applications for probiotics is to promote GI health. In elite athletes, up to 15% may experience transient GI symptoms self-rated as “moderate severity” or worse (40). In some sports, such as running, the proportion of participants reporting similar symptoms is 30% (66, 67), and even as high as 50% during ultra-endurance races (68). There are numerous factors that could lead to athletes experiencing GI symptoms at rest, during exercise (with or without peri-exercise feeding), or post-exercise (Figure 3). While other strategies have been employed to attenuate GI symptoms associated with these factors, probiotics may also offer benefits to athletes. However, such a large and broad range of factors (e.g., nutrition, heat, and timing of meals) that can contribute to GI symptoms means that potential confounding factors should be considered.

When studying the effects of probiotic supplementation on GI symptoms in athletes, the key considerations are the symptoms to be monitored, and how the prevalence and severity of these symptoms are recorded. There is a large range of methodologies used in probiotic research. For example, 4, 7, 9, and 10-point Likert scales have all been used, each of which also use different descriptor terms for symptoms (69–72). Symptoms should be well-defined for participants using simple and clear language (73). In studies where symptoms are monitored continually during an exercise bout, both the cumulative scores and peak severity scores should be reported (74). This approach ensures it is possible to identify moderate symptoms, and account for their duration, but also identify severe symptoms that could lead to cessation of exercise. Finally, researchers should employ objective criteria for symptom prevalence, and how severity improvement or alleviation is measured. Cumulative scores can be compared between conditions, although it is important to establish criteria for clinical/practical significance to infer real-world benefit. Other investigators have examined the frequency of symptoms subjectively scored as “moderate” severity or worse [e.g., (45, 67, 75)]. Readers are directed to the following resources for examples of validated and commonly used tools/questionnaires (70, 73, 74, 76). Implementation of these may help reduce much of the heterogeneity in the current body of evidence. As a minimum, it is recommended that all researchers:

1) Consider the specific GI symptoms most relevant to the research question.
2) These GI symptoms are clearly explained to participants, and that those explanations are published.
3) The GI symptom scores/ratings are clearly anchored and contextualized (e.g., what score would be the lowest value attributed to impairment of exercise performance or quality of life).
4) For symptoms during exercise, symptoms scores are recorded throughout and then both cumulative and peak scores for each individual symptom are reported.

### Stress and Mood

The gut-brain axis is now recognized as a bidirectional pathway, encompassing the endocrine, immune, and central nervous systems (77). While capturing stress and mood outcomes can be complex, several studies have examined probiotic supplementation by employing validated questionnaires (78–81). Other investigators have examined biomarkers like cortisol when studying probiotics during stressful periods in college students (53, 82–84) and endurance athletes (85). To examine the effects of a symbiotic formulation in professional soccer athletes, validated questionnaires were used to assess mental health (86). In a more unique scenario, competitive football players were assessed to determine the effect of daily probiotic supplementation on anxiety-induced physiological parameters (54). Measurements were taken by a portable biofeedback device which recorded electroencephalography, heart rate, and electrodermal responses, in combination with cognitive tests. While this may not be feasible for every application, investigators targeting stress and mood-related outcomes could also measure pertinent biomarkers such as circulating epinephrine, norepinephrine, dopamine, serotonin, corticotropin-releasing hormone, and adrenocorticotropic hormone. Such measures could be paired with a validated questionnaire and, heart rate variability to increase the relevance to athletic performance. More established questionnaires such as the Beck's Depression Inventory (87), the State-Trait Anxiety Inventory (88), and Profile of Mood States (89), as well as more population-specific tools like the Acute Recovery and Stress Scale and the Short Recovery and Stress Scale (90, 91), can easily be utilized.

### Upper Respiratory Tract Infections

The GI tract is a major gateway for pathogen entry and heavily protected by the immune system. Interactions with the immune system to enhance defenses against URTIs is the potential benefit of probiotics for athletes that has been most extensively researched (4). URTIs have the potential to reduce training load, cause an athlete to miss training or competition, and ultimately decrease physical performance. As an outcome, simplistic self-reported or clinically-verified measures for the reduction in incidence, duration, and/or severity of symptoms from illnesses particularly URTI can be employed. These measures have been a main outcome in many immune and respiratory health surveillance studies in athletes (14, 46, 92–95). Investigators should monitor intervention during periods when athletes may be at increased risk, such as a time of increased training volume/intensity, travel, competitions, compromised nutrient intake (e.g., decreased carbohydrate intake), or certain seasonal time frames like winter and the pollen season. Tracking training volume or training days missed due to illness (time loss) may be useful for interpretation of illness data. Questionnaires tailored specifically for quantifying illness symptoms in athletes are available, and we direct interested readers to the following resources (96, 97).

### Exercise Performance and Recovery Measures

Outcomes related to exercise performance are becoming more common in athlete/exercise focused probiotic trials, although there is large heterogeneity in experimental approaches and methodology. This scenario is likely due to the various physiological pathways that particular strains may be promoting performance improvements. For example, *L. plantarum* TWK10 increased time to exhaustion by ~8 min (treadmill exercise at 85% maximal oxygen uptake) and elevated blood glucose concentrations following exercise-to-exhaustion after 6 weeks of high dose (1 × 10^11^ CFU) supplementation in healthy male adults, compared to placebo (98). This strain can also improve energy harvesting capacity of skeletal muscle (99), which may account for enhanced exercise performance. Other studies suggest that improvements in performance are accompanied by reduced risk and/or impact of URTI. Individuals with fewer episodes of infections such as a common cold are typically able to train more often and harder (4). Finally, another indirect mechanism for increasing performance is related to improving muscle recovery. Probiotic supplementation may promote improved dietary protein absorption and utilization, which may improve performance measures (53, 57, 100). Therefore, outcome measures for performance should be selected based on the proposed mechanism of action of the strain. There is a wide range of validated performance measures that researchers may consider.

**Key Recommendations:**

- For GI symptoms, there are many confounding factors that should be accounted and controlled for where appropriate and feasible in RCTs. Psychological stress, habitual dietary intake, underlying pathology, and exercise environmental factors can all affect the likelihood of GI symptoms, and so should be considered and controlled.
- Careful consideration should also be given to how GI symptoms will be measured, how the severity and prevalence are assessed, and to ensure that it is possible to detect clinically meaningful differences between conditions.
- Measuring stress reduction and mood is difficult. Validated questionnaires are recommended, and where feasible, other measures such as biomarkers, heart rate variability, and sleep quality can be used to complement these data.
- URTIs and related illnesses can be quantified by incidence, duration, and/or severity of symptoms, and paired with biochemical immune response markers as a secondary outcome.
- Outcome measures for performance should be specific to the demands and requirements of the sport. The probiotic under study should support these outcomes, based on the proposed mechanism of action of the strain.

## Secondary Outcomes

### Changes in Microbiome

In many probiotic-related studies, researchers will measure discrete aspects of the GM. The human GM is defined as the community of microorganisms colonizing the GI tract containing thousands of different bacterial taxa as well as various archaea, eukaryotic microbes, fungi, and viruses (101, 102). Depending on the location in the GI tract, these microorganisms may play an important role in nutrient uptake, vitamin synthesis, energy harvest, mood regulation, inflammatory modulation, and host immune response (103). Studies of the athlete GM in recent years have identified distinct differences compared to non-athlete controls. In the first human athlete-focused study, rugby players were shown to have a more diverse microbiome than age- and body mass-matched controls at both a functional and taxonomic level (56, 59). Gut microbial diversity has been mooted as a proxy for health, and fitness levels have been positively correlated with microbial diversity (104, 105). Various studies indicate that athletes have greater levels (naturally) of specific taxa including, *Prevotella* (58), *Methanobrevibacteria* (58), *Akkermansia* (56), and *Veillonella* (31). However, the type of sport an athlete participates in can shape their microbiome, a factor which needs to be considered when investigating probiotics (106).

Advances in sequencing technologies, and substantial reductions in associated costs in recent years, has made microbiome sequencing more accessible. GM sequencing is commonly either compositional (“who is there?”) or functional (“what can they do?”) metagenomics, or more infrequently metatranscriptomics (“what genes are regulated/activated?”). Compositional metagenomics or amplicon sequencing amplifies a target gene (e.g., 16S rRNA for bacteria or ITS for fungi) and returns the taxonomical profile of a community. Functional or shotgun metagenomics randomly shears the entire DNA of the community, and returns not only taxonomy but also the functional profile of the community. Functional metagenomics, while generating more data, is more expensive and harder to analyze than compositional metagenomics. Metatrascriptomics sequences the rRNA of the community returning the functional genes, but is technically difficult, needs fresh fecal samples, and is expensive. The limiting factor in all of the methods is the selection of downstream data analysis tools and database accuracy, i.e., selecting different analysis tools can result in different outcomes, hence consistency of analysis tools is key (107, 108). More targeted approaches using quantitative polymerase chain reactions (qPCR), are especially applicable to probiotic studies, in combination with overall gene surveys, as they help complete the viability piece. As researchers are encouraged to test the viability of the probiotics as inputs, so might they test outputs from the stool to confirm that the probiotic actually (transiently) “colonized” the gut. Follow-up periods can also test the probiotics persistence after discontinuing use to quantify how long the probiotic strains remain detectable in the stool (20). It should be noted that strain recovery is not a measure that is necessarily associated with certain GM measures (e.g., diversity) and there may be large variability between individuals (20). Additionally, it is helpful to evaluate whether the probiotic had an effect on the overall GM community structure and metabolism (*via* fecal metabolomics). These are all important considerations not often tested/reported in the current body of probiotic literature. Therefore, taking pre- and post-measurements of the GM is recommended.

### Immune and Inflammatory Markers

Beyond modulating the GM, probiotics can induce local anti-inflammatory effects through immune system stimulation. The intestine contains GALT where the majority of immune cells are localized ensuring tight junction regulation and host defense. Furthermore, the common mucosal immune system enables lymphocytes primed within the immune system to circulate to distal sites, including the respiratory tract. Constantly challenged with antigens, commensal and opportunistic microorganisms, as well as potential pathogens, GALT works with the GM to manage invaders, and ultimately plays a large role in the downstream effects of the native and adaptive immune systems (109). Orally consumed probiotics may effectively “flood” the upper small intestine with bacteria (e.g., ~ 10^8−9^/gm) temporarily overwhelming the resident microbial population (~10^4−7^/cm^2^) during the transient passage of these exogenous bacteria through the GI-tract. Given the dynamic changes constantly occurring at the mucosal surface of the small intestine, such as variable mucin production and accumulation, orally delivered microbes are more likely to have greater access to the intestinal mucosa, microvilli, Peyer's Patches, and dendritic cells, to signal the immune system (110). Therefore, potential immune responses may be important secondary outcomes to measure, particularly when researchers are studying primary outcomes such as the prevention of URTIs [e.g., (94)]. Immune response and the methodologies used to measure it are complex. Researchers should use targeted approaches, based on mechanistic data and other clinical research [for reviews see: (111, 112)]. Additional work is needed to examine how probiotics affect both the acute inflammatory response to exercise, and resting inflammatory control during times of increased environmental, social, or physical stress in athletes.

**Key Recommendations:**

- When feasible, measures of the GM, including compositional and/or functional data, are recommended at least pre- and post-supplementation in probiotic trials.
- More targeted approaches using qPCR may be of great value to researchers who wish to investigate persistence of an administered probiotic.
- Measurement of circulating and salivary immune markers is encouraged for studies on probiotics to identify purported mechanisms, and investigating primary outcomes such as URTIs.

## Safety Data

Collecting and reporting safety data during probiotic studies is greatly lacking in trials with athletic populations (4). Probiotics may theoretically be responsible for four types of side effects, including systemic infections, adverse metabolic effects, cytokine-mediated immunologic events in susceptible individuals, and transfer of antibiotic resistance genes (113). While healthier individuals including athletes may be at lower risk for these issues than other populations, rigor and transparency is a fundamental requirement. Data including adverse events and serious adverse events are important to host organizations, funding agencies, institutional review boards, other researchers, consumers, and national regulatory agencies, in addition to the research participants involved. For example, athletic consumers may be interested to know if a particular probiotic might induce minor GI symptoms. Moreover, studies detailing safety data in athletic populations can also inform new areas in relevant clinical populations. These effects can easily be monitored in research settings with questionnaires covering issues including abdominal cramping, nausea, soft stools, flatulence, and taste disturbance (113). While research articles may not explicitly state these issues as “adverse events,” they may be reported, inadvertently, depending on the research outcome, for example, if an investigator is researching GI symptoms. Regardless, adverse events and negative side effects should be reported and we encourage investigators to use this terminology explicitly.

**Key Recommendations:**

- Safety assessments should be performed and reported for all probiotic trials.

## Strengths and Limitations

In many instances, strengths and limitations of experimental designs and methodologies are not adequately reported in athletic-focused probiotic trials. This is a component generally included in quality assessments when conducting systematic reviews and meta-analyses. Such information is not only important for transparency of the published article, but also for efforts to collate and synthesize findings in future review studies. One example that has emerged is use of controlled laboratory investigations (probiotic efficacy) compared to more specific “sport applications” in an athlete's natural environment (probiotic effectiveness). Laboratory-controlled trials have generally set stricter limits on the type of training. Some studies have employed extreme exercise bouts to; (1) examine the effect of co-administration of protein and probiotics on muscle damage, recovery and performance outcomes (114), (2) evaluate the effects of probiotic supplementation on GI permeability when exercising in the heat (115), and (3) evaluate time-to-fatigue (116). In these types of studies, participants are often non-athletes, though they may have been physically active and healthy. Examination of athletes in their normal training environment have included competitive cyclists (34), male and female Division I athletes following an offseason resistance training program (16, 53), marathon runners (45), and female Division I swimmers (33). In these studies, athletes continued their normal training regimen. To recruit athletes into trials that deviate from their planned training is difficult, and likely not feasible at high levels. This is clearly a limitation and investigators will have difficulty enrolling athletes into strict regimens that substantially deviate from their normal training program. More work is needed in both areas, but the strengths and weaknesses of these designs should be highlighted along with the specific methodology, type of participant, study context, and external validity. Table 1 can be used to identify strengths and limitations of existing, ongoing, and future studies.

Finally, investigators should register their clinical trial with a national registry such as clinicaltrials.gov before commencing their study and implement a RCT checklist, such as Critical Appraisal Skills Programme (CASP) (117). Moreover, investigators should adhere to established reporting guidelines such as the Consolidated Standards of Reporting Trials (CONSORT) (118). This process not only fulfills ethical obligations to participants and the research community, but also provides information to potential participants and referring clinicians, reduces publication bias, assists editors and others in understanding the context of study results, promotes more efficient allocation of research funds, and helps institutional review boards (IRBs) determine the appropriateness of a research study (119).

**Key Recommendations:**

- Research teams should ensure strengths and limitations are described in project reports and submissions to a peer-reviewed journal.
- Researchers should meet institutional review board requirements on the conduct of research particularly related to allocation concealment, randomization, bias, and relationships with probiotic manufacturers.
- Investigators should register their trial with a registry such as clinicaltrials.gov before commencing their study.

## Considerations for Probiotic-Related Preparations

### “Dead on Arrival”: The Issue of Paraprobiotics (Aka: Inactivated Probiotics)

There is an increasing interest in supplementation with non-viable microorganisms or microbial cell extracts used in a similar manner as probiotics. Preparations from certain probiotic strains (such as lactic acid bacteria and bifidobacteria) have shown to maintain health benefits even after being no longer viable (120). A review of 40 randomized controlled trials indicated paraprobiotics were not more effective when compared to identical living probiotic strains in most cases, but in 15% of treatment trials, dead microbes were found to be effective, thus showing there may be a role for paraprobiotics (121). Further, these non-viable preparations may overcome many of the drawbacks of traditional probiotics such as storage requirements and shelf-life considerations. Favorable properties of heat-killed bacteria have been observed *in vitro* (122), in animal models (123), in human trials (124, 125), and more recently in athletes (126). These preparations have been studied for beneficial health effects and may be a potential solution for overcoming stability problems. However, live probiotics appear to have some superior efficacy to heat-inactivated microbes for both *in vitro* and clinical studies (127–129). The difference in results may also be related to differing participant health characteristics and study outcome measures that are uniquely impacted by probiotics vs. paraprobiotics.

The presence of dead or injured microbes in commercial probiotic products is unavoidable, as some death occurs during product transport and storage. The standard approach to maintaining the target dosage of live probiotics entails addition of surplus probiotics to account for any death that might occur during storage. While paraprobiotic products do not need to account for organism loss, there is still a need to report the method of inactivation, evaluate their stability and activity across their designated shelf life, and use robust and repeatable methods to assess their biological effects. In addition, comprehensive safety information should also be explored, even though these preparations have been theorized to present less safety concerns (120).

### Synbiotics

Pairing prebiotics with probiotics, known as synbiotics, purportedly increases the overall effectiveness of a preparation, as the prebiotic component may promote the growth and activities of probiotic component (130). Synbiotics are defined by the International Scientific Association for Probiotics and Prebiotics (ISAPP) as, “a mixture comprising live microorganisms and substrate(s) selectively utilized by host microorganisms that confers a health benefit on the host” (131). Synbiotic studies are becoming increasing prevalent in athlete-related investigations and have yielded positive results (51, 86, 132, 133). An expert panel from the ISAPP has published a Consensus Statement detailing the levels of evidence (existing and required), safety, effects upon targets and implications for stakeholders of the synbiotic concept (131). Ideally, the health benefit of a synbiotic would be super-additive, that is, it would exceed the benefit observed for the sum of the individual components. The ISAPP stated that a product containing a probiotic and a prebiotic that only has evidence for each component individually, and not as a combination product, should not be called a synbiotic. There should also be at least one appropriately designed study of the synbiotic in the target host that demonstrates both selective utilization of the substrate and a health benefit (131). As with probiotics, the safety, identity, purity, and potency of the live microorganism should be clearly and accurately described according to the best available methods. Testing should meet applicable regulatory standards for the product category, and the structure and purity of the substrate stated and characterized by appropriate chemical analyses (131). The matrix in which the probiotic component is incorporated, such as a power or liquid, may present viability challenges. Coupled with the manufacturers ensuring viability, stability testing, and the appropriate overages, the investigator should also consider testing viability through the intervention period. Correct storage conditions also need to be conveyed to study participants during usage as water exposure and temperature extremes may impact the integrity of the preparation. Given the main mechanism of action of synbiotics is to induce an increase in the survival of probiotics in the host, investigators should consider enumerating the microbial strains during the intervention *via* qPCR.

### Postbiotics

In 2019, the ISAPP defined postbiotics as a “preparation of inanimate microorganisms and/or their components that confers a health benefit on the host” (134). Postbiotics include the metabolic substrates produced by probiotic bacteria. Several studies indicate that these metabolic substrates modulate the intestinal epithelial/mucosal immune system (135) and may have clinical benefits (136). It is essential that the inanimate microorganisms and metabolites in postbiotic preparations are well-characterized, and targeted toward specific parameters of the host to induce a clinical benefit. We are at this stage unaware of any research examining the use of postbiotics in athletes.

**Key Recommendations:**

- Research in paraprobiotics, synbiotics, and postbiotics in athletes should follow similar reporting standards as probiotics, including safety information.
- Paraprobiotics, postbiotics, and synbiotics are not probiotics by the accepted definition, and require additional study design and reporting considerations. Planning athlete trials should have a strong justification for using these products.

## Conclusion

The increasing interest in probiotics for athletic applications is evidenced by the growing number of published works and trial registrations. Research has produced promising results in GI health, exercise performance and recovery, physical fatigue, immunity, and body composition. However, the heterogeneity of human clinical trials reporting probiotic use in these applications presents additional and unique challenges regarding interpretation, comparison, and, ultimately, the utility of these studies (5). In general, studies should adhere to standard human trial design and reporting guidelines (137) and best practices for GM research (138). Here we have presented a framework of best practices specific to athlete-focused investigations with exercise (or sport) performance, prevention of common illnesses, health, and mechanistic outcomes. Key recommendations raised may also be considered in the context of pre-clinical and/or early phase investigations, which are an important component of a clinical development pipeline. Researchers need to clearly define the planned probiotic intervention, implement the appropriate study design, participant selection, and establish outcomes aligned with the research question. In addition, accurately reporting safety data and being transparent with the strengths and limitations is recommended, as these elements are often lacking in athlete-focused probiotic research. Given the substantial time, effort, and expense invested in probiotic trials, ensuring consistent, valid, and transparent approaches to assessing the effectiveness of these preparations is important. Properly reporting, presenting, and communicating the results will enhance the quality of evidence and value of practical applications of probiotic supplementation in athletes.

## Author Contributions

AM: conceptualization. AM, JP, and LM: methodology, literature review, and project administration. AM, JP, OO'S, KB, JT, DP, FW, NW, CW, and LM: writing—original draft preparation and writing—review and editing. AM and JP: visualization. LM: supervision. All authors contributed to the article and approved the submitted version.

## Conflict of Interest

AM is employed by Isagenix International LLC. Isagenix was not involved in any aspect of the review. JP is a consultant for Aliment Nutrition Ltd. JT has previously received grants to evaluate the efficacy of various nutritional supplements including probiotics. FW received research funding from Royal Friesland Campina N.V., Amersfoort, the Netherlands after this review was drafted. NW has been the recipient of research from DuPont, Chr Hanson, Yakult, Probiotics Australia, UAS Laboratories, and Winclove Probiotics. CW serves on scientific advisory boards for the Wheat Foods Council, Ardent Mills, LLC, and the Hass Avocado Board Avocado Nutrition Science Advisory group. LM is on the Scientific Advisory Board for Bio-K+, Canada and on the Microbiome Advisory Board for Biocodex, France and is a paid lecturer for both companies. The remaining authors declare that the research was conducted in the absence of any commercial or financial relationships that could be construed as a potential conflict of interest.

## Publisher's Note

All claims expressed in this article are solely those of the authors and do not necessarily represent those of their affiliated organizations, or those of the publisher, the editors and the reviewers. Any product that may be evaluated in this article, or claim that may be made by its manufacturer, is not guaranteed or endorsed by the publisher.

## Abbreviations

BSH: Bile salt hydrolase
CFU: Colony forming units
FFQ: Food frequency questionnaire
GALT: Gut associated lymphoid tissue
GI: Gastrointestinal
GM: Gut microbiome
HEI: Healthy eating index
ISAPP: International Scientific Association for Probiotics and Prebiotics
IRB: Institutional review board
qPCR: Quantitative polymerase chain reaction
RCT: Randomized controlled trial
URTI: Upper respiratory tract infection.
